# Glial fibrillary acidic protein in fibromyalgia: its serum levels and antibodies

**DOI:** 10.1186/s42358-026-00552-0

**Published:** 2026-06-04

**Authors:** Felipe Massó, Laura-Aline Martínez-Martínez, Luis M. Amezcua-Guerra, Francisco Mercado, Angélica Almanza, Manuel Martínez-Lavín

**Affiliations:** 1 Unidad de Investigación en Medicina Traslacional, Instituto Nacional de Cardiología Ignacio Chávez, Mexico City, Mexico; 2 Departamento de Reumatología, Instituto Nacional de Cardiología Ignacio Chávez, Mexico City, Mexico; 3 Departamento de Inmunología, Instituto Nacional de Cardiología Ignacio Chávez, Mexico City, Mexico; 4 Laboratorio de Fisiología Celular, Dirección de Investigaciones Biomédicas en Salud Mental, Instituto Nacional de Psiquiatría Ramón de la Fuente Muñiz, Mexico City, Mexico

**Keywords:** Fibromyalgia, Glial fibrillary acidic protein, GFAP, Satellite glial cell, Dorsal root ganglia, Neuropathic pain

## Abstract

**Background:**

Fibromyalgia is a stress-related disorder in which dorsal root ganglia (DRG) may play an important pathogenic role. DRG exhibit unique stress-induced, pro-algesic physio-anatomy, where each pain-sensing nerve fiber soma is encased and interacts with immune-competent satellite glial cells (SGCs). Patients suffering from fibromyalgia harbor anti-SGCs antibodies; however, the specific SGCs antigen(s) remain unidentified. Glial fibrillary acidic protein (GFAP) is an intermediate filament protein serving as early SGCs activation marker. Different environmental stressors induce GFAP upregulation and structural modifications, including citrullination, potentially rendering it immunogenic. GFAP antibodies are implicated in autoimmune encephalomyelitis. We determine whether the serum of patients with fibromyalgia, collected before the COVID-19 pandemic, overexpresses GFAP and/or harbors antibodies against GFAP.

**Methods:**

We studied 47 women with fibromyalgia and 31 healthy women. For GFAP antibody detection, a sensitive ELISA was developed using recombinant human GFAP. A commercial human GFAP ELISA Kit was used to measure GFAP serum levels.

**Results:**

Significantly higher serum GFAP antibody optical density (OD) was detected in patients with fibromyalgia (median 0.04, 0.02–0.10 vs. 0.02, 0.01–0.05; *p* = 0.025). When the control group 99th percentile value (0.127 OD) was used as positive threshold, 9/47 (19%) patients tested positive for anti-GFAP antibodies. Patients with fibromyalgia showed numerically higher GFAP serum levels: (244.0 pg/ml ± 82.5 SD versus 211.4 ± 65.2. *p* = 0.057).

**Conclusion:**

In this proof-of-concept study, patients suffering from fibromyalgia exhibit higher serum anti-GFAP antibodies and numerically augmented circulating GFAP levels. Future mechanistic studies will define GFAP role in the pathogenesis of FM.

## Background

A longstanding line of investigation proposes fibromyalgia (FM) as a stress-evoked, sympathetically maintained neuropathic pain syndrome. This hypothesis situates dorsal root ganglia (DRG) at the epicenter of FM pathogenesis [1–2]. The DRG house the soma of nerves conveying painful stimuli from the body surface and from internal organs, and there is a clear relationship between FM and small-fiber neuropathy. The DRG lie outside the blood‒brain barrier but remain shrouded by meningeal layers and are bathed in the cerebrospinal fluid. Blood-borne molecules, antigens, antibodies and infectious agents can gain access to these ganglia. In the DRG, each pain-sensing nerve fiber soma is tightly encased and interacts with several metabolically active, immune-competent satellite glial cells (SGCs) [3].

SGCs may play a major role in stress-evoked neuropathic pain. After peripheral nerve injury, there are cellular plasticity responses of SGCs in the DRG leading to neuropathic pain [4]. SGCs also envelop the neuronal soma of the paravertebral sympathetic ganglia, and sympathetic dysfunction is prevalent in FM [3].

Recent research [5.6] suggests that DRG-SGCs may play an important role in the pathogenesis of FM: Mice receiving IgG from patients with FM display mechanical and cold hypersensitivity and small nerve fiber pathology; in these instances, IgG is exclusively deposited in mouse SGCs [5]. A subgroup of individuals suffering from severe FM harbors anti-SGC antibodies [6]; nevertheless, the purported FM-related SGC antigen(s) remain unidentified.

Glial fibrillary acidic protein (GFAP) is an intermediate filament protein serving as an early SGC activation marker. Different environmental stressors induce GFAP overexpression and conformational changes, including citrullination [7], potentially rendering it immunogenic. GFAP up regulation is associated with the release of pronociceptive mediators such as cytokines, chemokines, and growth factors, which modulate neuronal excitability and pain hypersensitivity [8]. GFAP antibodies have been implicated in the development of different autoimmune encephalomyelitis syndromes [9]. GFAP serum high levels reflect glial cell activation [10].

The COVID-19 pandemic has left many previously healthy individuals with persistent FM-like symptoms. This post-COVID-19 condition is also associated with anti-SGC antibodies [11] and may introduce a confounding factor to FM pathogenic studies.

The objective of this proof-of-concept study was to define whether the serum samples of patients suffering from FM, which were collected before the COVID-19 pandemic, overexpress GFAP and/or harbor antibodies against GFAP.

## Methods

### Patients

We included 47 women with FM satisfying the following inclusion criteria: aged 18–50 years, fulfilling the Wolfe et al. 2016 FM diagnostic criteria, disease duration of more than 2 years and pain intensity no less than 4/10 on a visual analog scale. The exclusion criteria were obesity and concurrent metabolic, autoimmune, neoplastic or neurological diseases. The control group comprised 31 age-, sex-, and BMI-matched healthy individuals. The same exclusion criteria were applied to the control group. A rheumatologist expert on FM examined each case to corroborate the FM diagnosis or the healthy status of the controls.

All participants provided written informed consent and completed standardized questionnaires, including the 2016 Wolfe et al. criteria, FIQ-R, COMPASS-31, Small Fiber Symptom Survey, S-LANSS, PHQ-9, GAD-7, IPAQ, and EuroQol-5D. Blood samples were collected between 2017 and early 2019, before the COVID-19 pandemic. Samples were obtained in the morning after an overnight fast, and serum and plasma aliquots were separated immediately and stored at -70 °C. This exploratory study used a convenience sample of all available pre-COVID-19 sera, and no prior sample size calculation was performed. Only women were included to reduce potential sex-related confounding. The protocol was approved by the Ethics and Research Committees of the National Institute of Cardiology of Mexico (INCAR-DG-DI-CI-DICT-023-2021).

We developed a sensitive ELISA test to search for serum GFAP antibodies: 96-well ELISA plates were coated with 20 ng of GFAP per well (Abcam Waltham, MA, USA) in 0.05 M carbonated buffer (pH 9.1). We assayed several amounts of GFAP (10–100 ng per well) to obtain optimal concentration. Plates were washed with PBS-0.3% Tween 20, blocked with 1.0% casein in PBS for 2 h and washed again. Then, 100 µl of the participants’ serum were incubated overnight. After further washing, 100 µl of anti-human IgG-HRP antibody (Abcam, Waltham, MA, USA) were added, and the mixture was incubated for 1 h at room temperature. The microplates were washed again and color-developed with O-phenylenediamine-H2O2 in citrate/phosphate buffer for 30 min; the reaction was stopped by adding 50 µL of 0.2 M H2SO4. The plates were read in a Cytation 3 ELISA reader (Agilent Santa Clara, CA) at 495 nm. We included a positive control (a duplicate of a 20 ng GFAP-coated well reacting with a commercial monoclonal anti-GFAP antibody) (Abcam Waltham, MA, USA) in each plate. Sera were run in duplicate, and the operator was blinded to the serum provenance (patient or control). Intra-assay coefficient of variation (CV) was 3.5% whereas inter-assay CV was 8.7%. To ensure specific reactivity to human serum antibodies, we selected a commercial human recombinant protein providing a pure, contaminant-free, ELISA well-plate binding protein.

A commercial Thermo Fisher Human GFAP ELISA Kit (Invitrogen EEL079, Thermo Fisher Scientific Inc., USA) was used to measure GFAP serum levels according to the manufacturer’s instructions.

### Statistical analysis

Numerical variables with a normal distribution are presented as means ± standard deviation (SD), while non-normally distributed variables are reported as medians with interquartile ranges (IQR). The Kolmogorov-Smirnov test was used to assess the normality of data distribution. Intergroup comparisons were performed using the Mann-Whitney *U* test, the chi-square test, or Fisher´s exact test, as appropriate. Sperman test was used to correlate serum anti-GFAP antibody level and serum GFAP concentration. To establish a positivity threshold, the 99th percentile of the distribution of values observed in the control group was calculated. A *p* value less than 0.05 was considered statistically significant. Analyses were performed using SPSS software, version 23.0 (IBM Corp, Armonk, NY, USA).

## Results

The outstanding demographic features of patients and controls are shown in Table 1. Both groups had similar age ranges and body mass indices. As expected, there was a marked difference in all the clinimetric parameters between patients and controls.

**Table 1:** Demographic features, clinimetric parameters, serum levels and antibodies against glial fibrillary acidic protein in women suffering from fibromyalgia and age/sex-matched controls

		Fibromyalgia *n* = 47	Control *n* = 31	*p*
Age (years)		42 ± 9	40 ± 9	0.368
Body mass index (Kg/m^2^)		25.9 ± 3.9	25.3 ± 3.1	0.690
Disease duration (years)		8 (5–12)	-	NA
Widespread pain (VAS 0–100)		76 (62–89)	3 (0–12)	< 0.0001
Tender points (0–18)		15 (14–18)	3 (1–4)	< 0.0001
Widespread Pain Index score		13 (9–17)	2 (1–3)	< 0.0001
Symptoms Severity Scale score		9 (7–10)	2 (1–4)	< 0.0001
Polysymptomatic Distress Scale score		21 (17–26)	4 (3–7)	< 0.0001
Revised Fibromyalgia Impact Questionnaire score		57 (34–70)	5 (2.5–9.3)	< 0.0001
Function domain		14 (7–20)	0 (0-0.7)	< 0.0001
Overall impact domain		12 (4–16)	0	< 0.0001
Symptoms domain		32 (20–36)	3.5 (1.5-9)	< 0.0001
Pain		7 (4–9)	0 (0–1)	< 0.0001
Lack of energy		7 (5–9)	1 (0–2)	< 0.0001
Stiffness		6 (2–8)	0	< 0.0001
Poor sleep quality		9 (6–10)	1 (0–3)	< 0.0001
Depression		5 (0–7)	0 (0–1)	< 0.0001
Memory problems		6 (4–8)	1 (0–3)	< 0.0001
Anxiety		5 (3–8)	1 (0–2)	< 0.0001
Tenderness to touch		7 (3–8)	0	< 0.0001
Balance Problems		5 (0–7)	0 (0–1)	< 0.0001
Sensitivity to loud noises, bright lights, odors, and cold		8 (3–10)	1 (0–2)	< 0.0001
Autonomic symptoms (COMPASS31)		41 (26–53)	12 (6–21)	< 0.0001
Orthostatic domain		20 (12–24)	4 (0–8)	< 0.0001
Vasomotor domain		1.67 (0-2.5)	0	< 0.0001
Secretomotor domain		6.4 (2.1–8.5)	0 (0-2.14)	< 0.0001
Gastrointestinal domain		8.9 (8-14.2)	4.4 (2.6–5.4)	< 0.0001
Bladder domain		1.1 (0-3.3)	0 (0-1.1)	< 0.012
Pupilar domain		3 (2.3-4)	1.6 (0.7-2)	< 0.0001
Neuropathic pain symptoms (S-LANSS)		19 (14–22)	0	< 0.0001
Small-fiber Symptom Survey (22-items)		34 (24–49)	6 (3–9)	< 0.0001
Small-fiber Symptom Survey (32-items)		52 (35–68)	8 (4–14)	< 0.0001
Gastrointestinal component		8 (6–11)	2 (1–3)	< 0.0001
Somatosensory component		7 (4–11)	0 (0–1)	< 0.0001
Miscellaneous component		10 (8–13)	2 (1–3)	< 0.0001
Microvascular component		7 (3–11)	1 (0–2)	< 0.0001
Urological component		1 (0–4)	0	< 0.001
Widespread Chronic Pain intensity (VAS 0–10)		8 (7–9)	1 (0–2)	< 0.0001
PHQ9		12 (8–17)	2 (1–5)	< 0.0001
GAD7		9 (6–14)	2 (1–4)	< 0.0001
EuroQol Health thermometer (VAS 0–100)		60 (40–75)	90 (85–95)	< 0.0001
International Physical Activity Questionnaire	Mild	10 (21.3)	8 (25.8)	
	Moderate	16 (34)	7 (22.6)	0.552
	High	21 (44.7)	16 (51.6)	
Anti-GFAP antibody serum levels (optical density) GFAP serum levels (pg/mL)		0.042 (0.025–0.109) 244.0 +/- 82.5	0.027 (0.019–0.051) 211.4 +/- 65.2	0.025 0.057

**Fig. 1:**
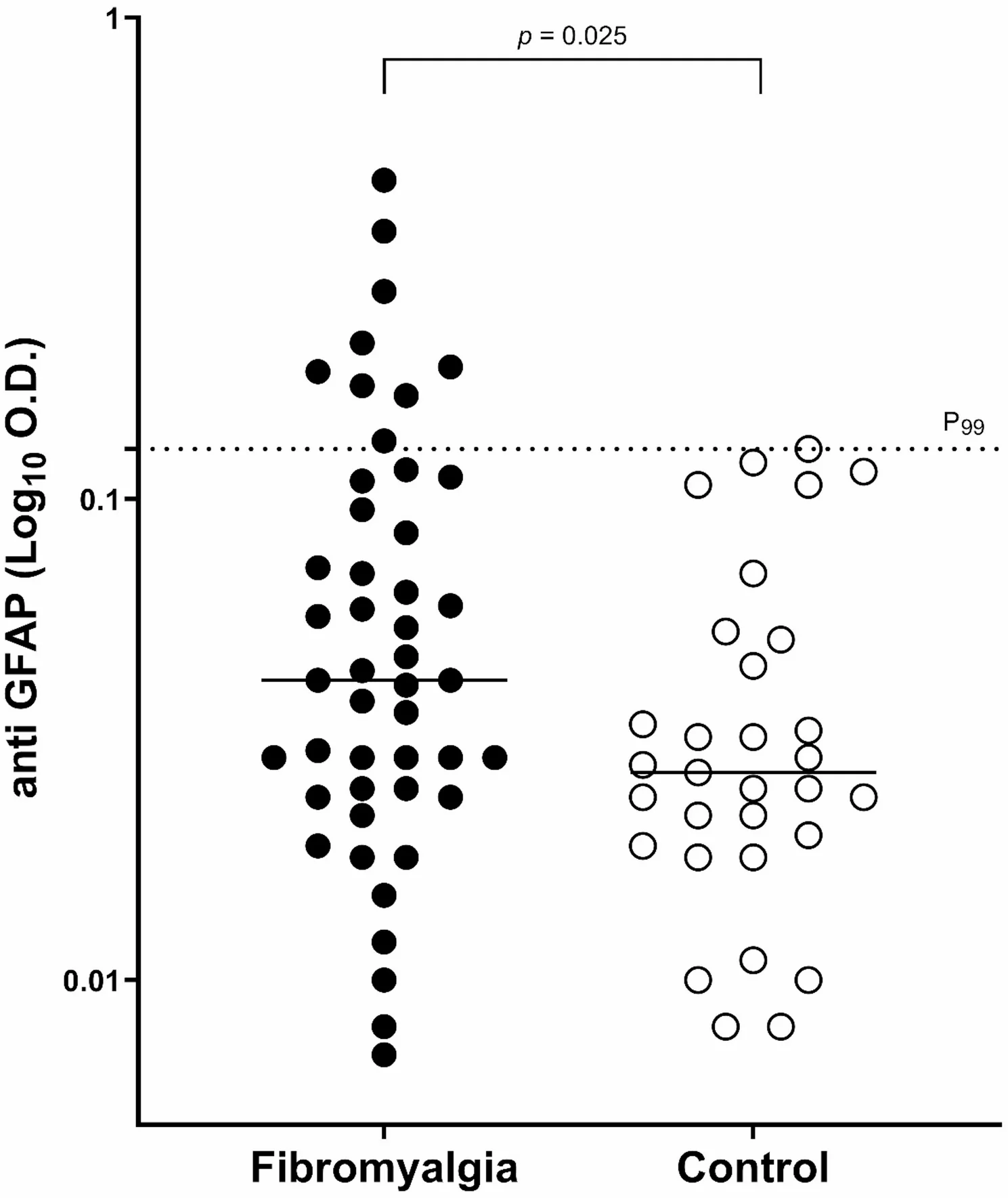
Base 10 logarithmic (Log10) depiction of anti-glial fibrillary acidic protein antibodies serum levels measured as optical density (O.D.) in patients with fibromyalgia and controls. The control group 99th percentile value was used as the cutoff point for antibody positivity. Solid lines define median values

Compared to controls, patients with FM presented greater GFAP antibody optical density (OD) (median 0.04, 0.02–0.10 vs. 0.02, 0.01–0.05; *p* = 0.025). When the control group 99th percentile value (0.127 OD) was used as the cutoff point, 9/47 (19%) patients suffering from FM tested positive for anti-GFAP antibodies (Fig. 1). Clinimetric scores measuring the severity of FM, autonomic dysfunction, or neuropathy were not different between GFAP antibody-positive and GFAP antibody-negative patients.

Patients with FM had numerically higher serum levels of GFAP (244.0 pg/ml ± 82.5 SD) than controls did (211.4 ± 65.2), yielding a *p* value of 0.057. There was no correlation between GFAP levels or GFAP antibody values with the different clinimetric parameters.

## Discussion

Our results revealed that, compared with healthy women, the serum samples of women suffering from FM, which were collected before the COVID-19 pandemic, exhibit statistically significant higher quantity of antibodies against GFAP as well as numerically augmented circulating GFAP levels (*p* = 0.057). Furthermore, 19% of patients have serum GFAP antibody concentration above the 99th percentile of controls. We use the 99th percentile of controls as anti-GFAP antibody positivity cutoff point to establish a stringent definition of abnormal reactivity to maximize specificity and minimize false-positive results. This antibody positivity ratio yields a sensitivity of 19%, specificity of 97%, positive predictive value of 90%, and negative predictive value of 44%. These numbers only reflect sample-specific ratios of this case-control study and cannot be generalized to the true population.

Normal serum GFAP levels depend on age, sex, and assay platform. In our study, the mean serum GFAP concentration in healthy individuals (211.4 ± 65.2 pg/mL) is similar with the values reported in the Thermo Fisher GFAP ELISA User Guide (mean 182.5 pg/mL).

In our in-house assay, the possibility of cross-reactive antibodies with other intermediate filament proteins seems unlikely. Recombinant Human GFAP protein (Abcam 114149) does not exhibit any other protein in the purity assay provided by the seller, and no other human protein was present on the ELISA well plate.

Concurrent antibody-positive autoimmune illnesses were excluded from our FM patient population making unlikely a GFAP antibody false-positive result. Our ELISA lacks epitopes detecting specific antibodies against citrullinated or other modified forms of GFAP.

Based on previous research on GFAP-associated disorders, we speculate that the percentage of GFAP antibodies in FM would be greater when the cerebrospinal fluid (CSF) of patients is analyzed. In the cohort described by Flanagan et al., among individuals with GFAP-associated autoimmune encephalomyelitis, 92% were GFAP-IgG positive in CSF, but only 45% were positive in serum [12]. However, intrathecal antibody production, blood-brain barrier protection and accessibility of circulating antibodies to their cognate antigen are likely different in autoimmune astrocytopathy versus FM. Our preliminary findings in serum samples may lead to much more complicated clinical studies searching for anti-GFAP antibodies in CSF of patients suffering from fibromyalgia and matched controls. Spinal tap is a required procedure in the clinical assessment of GFAP associated autoimmune encephalomyelitis, but not in cases of fibromyalgia.

Krock et al. reported that anti-SGC IgG serum levels were increased in patients with severe FM compared with healthy controls in two different cohorts. They did not define a cutoff point to calculate the percentage of patients with positive anti-SGC antibodies [6]. Our results suggest that GFAP could be one of the antigenic determinants of those anti-SGC antibodies. We found no difference in disease burden between GFAP antibody-positive and GFAP antibody-negative patients, tentatively arguing against a simple severity-driven immune mechanism for nociceptive sensitization. Larger studies will better define this issue.

The clinical features of FM differ from those of GFAP-associated autoimmune encephalomyelitis; a possible explanation for this variance could be the antigen location; DRG, in patients with FM versus the CNS in patients with autoimmune encephalomyelitis.

Traditionally, GFAP has been considered a structural protein without effector activity whose upregulation reflects glial activation in response to different stressors; nevertheless, genetic deletion of GFAP or pharmacological knockdown using antisense oligonucleotides suggests that GFAP not only serves as a marker for glial cell activation but also may play a role in the maintenance of neuropathic pain states [13]. The results of our investigation raise the possibility that GFAP may play a pathogenetic role in widespread FM pain.

FM and post-COVID-19 conditions display overlapping clinical features, including chronic fatigue, widespread pain, cognitive dysfunction, and sleep disturbances. They may also share pathogenetic mechanisms: small-fiber neuropathy, dysautonomia [14] and anti-SGC antibodies [11] have been described in both entities. These overlapping features may introduce confounding factors to FM pathogenetic studies taking place after the COVID-19 pandemic. Patients or controls participating in FM studies may have had abnormalities related to previous COVID-19 infection.

One strength of our study is that we used serum samples stored before the COVID-19 pandemic. On the other hand, this peculiarity is also a limitation preventing us from expanding the cohort size to define whether the difference in GFAP serum levels between patients and controls falls below the inflexible 0.05 *p* significance value. The exclusion of men and of patients with common FM comorbidities including obesity, metabolic or autoimmune diseases, limits the external validity of our study.

Seefried et al. recently reported that 13/68 (19%) of patients with fibromyalgia and 0% of healthy controls harbors serum antibodies against rat DRG citrullinated proteins [15]. The identity of the citrullinated proteins was unknown.

**Fig. 2:**
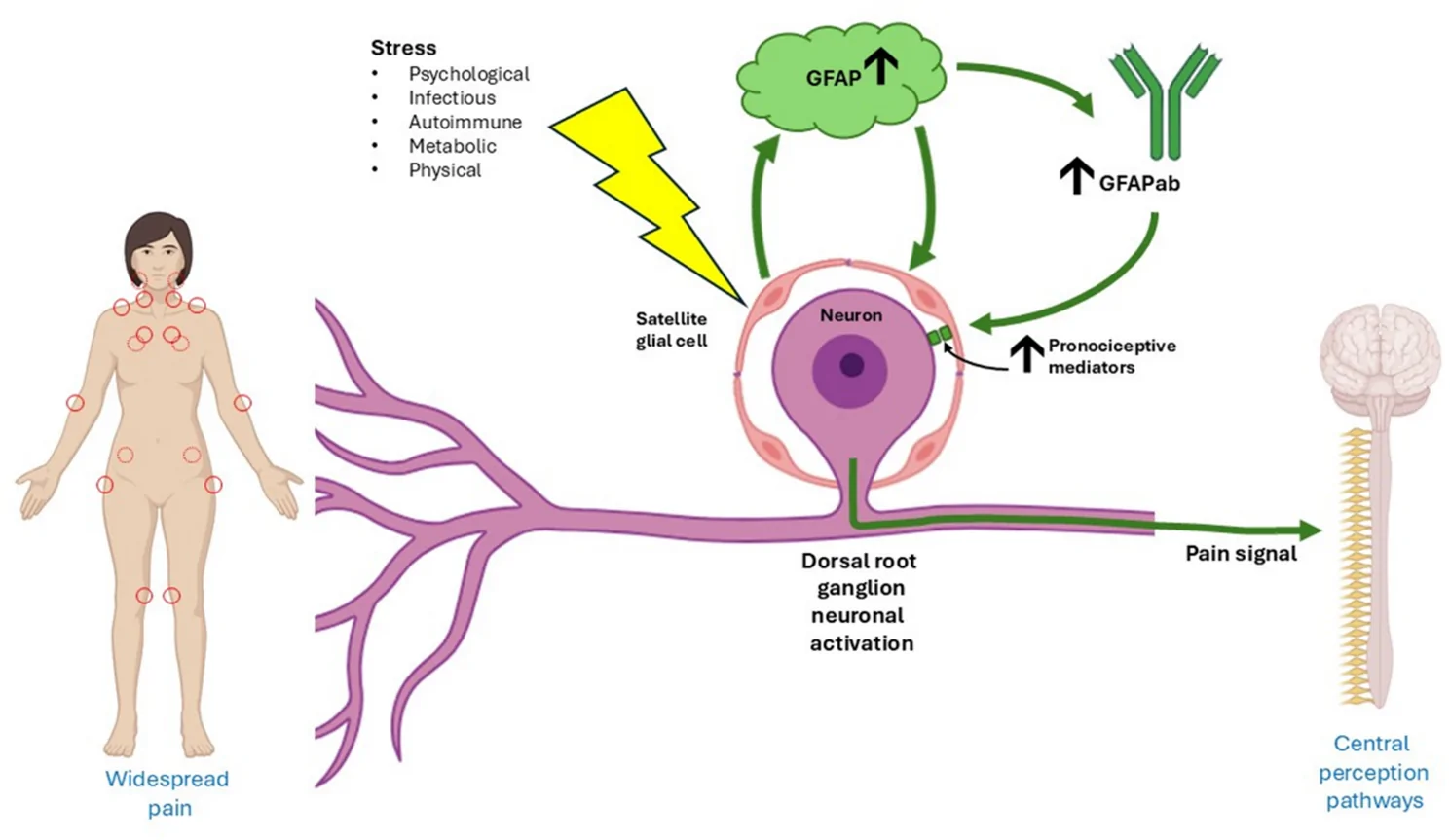
Preliminary, hypothetical pathogenic mechanisms for fibromyalgia based on recent knowledge and on the results of this study. Diverse stressors may activate dorsal root ganglia satellite glial cells (SGCs) with GFAP overexpression and conformational modifications inducing antibody response in a subgroup of patients. Activated SGCs release pronociceptive mediators that sensitize the encased nociceptive neuron soma, leading to widespread peripheral pain. (Image created with BioRender.com and Microsoft PowerPoint 360 version 2505)

Building on developing knowledge and on the results of the present investigation, we hypothesize the following mechanisms leading to FM (Fig. 2): different psychological, physical, infectious, metabolic and/or autoimmune stressors, can activate DRG-SGCs with GFAP conformational modifications, including citrullination and with GFAP overexpression. Modified GFAP may serve as novel antigen, triggering an antibody response in a subgroup of patients. GFAP overexpression and antibody formation could release pronociceptive mediators, sensitizing the encased DRG pain-transmitting nerve soma. The convergence of all peripheral pain-transmitting nerve fibers at the DRG could explain how nociceptive signals arising from the DRG are sensed as widespread peripheral pain (Fig. 2). Nevertheless: given the cross-sectional, case–control design, small sample size, and absence of functional or mechanistic data in our study, no causal association between GFAP and FM can be established at this time. These preliminary findings need larger replication cohorts, Western blot or immunohistochemistry assays, CSF analysis, and mechanistic studies to determine the real role of GFAP in the pathogenesis of FM.

## Conclusion

Our proof-of-concept study found that a subgroup of patients suffering from FM harbors antibodies against GFAP. These patients also display a tendency for increased serum concentration of GFAP suggesting glial cell activation. Future mechanistic studies will define the real role of GFAP in the pathogenesis of FM.

## Data Availability

The datasets used and/or analysed during the current study are available from the corresponding author on reasonable request.

## Abbreviations

DRG: Dorsal root ganglia
SGCs: Satellite glial cells
GFAP: Glial fibrillary acidic protein
OD: Optical density
FM: Fibromyalgia
